# A systematic review of the effect of sleep interventions on presenteeism

**DOI:** 10.1186/s13030-021-00224-z

**Published:** 2021-11-17

**Authors:** Yuta Takano, Suguru Iwano, Shuntaro Aoki, Norihito Nakano, Yuji Sakano

**Affiliations:** 1 Department of Psychology, Fukuyama University, 985-1 Sanzo, Higashimura-Cho, Fukuyama, Hiroshima, 729-0292 Japan; 2 Graduate School of Psychological Science, Health Sciences University of Hokkaido, 2-5 Ainosato, Kita-ku, Sapporo, Hokkaido 002-8072 Japan; 3 Faculty of Welfare and Health Science, Oita University, 700 Dannoharu, Oita City, Oita 870-1192 Japan; 4 Center for Medical Education and Career Development, Fukushima Medical University, 1 Hikarigaoka, Fukushima, 960-1295 Japan; 5 Department of Neuropsychiatry, Fukushima Medical University, 1 Hikarigaoka, Fukushima, 960-1295 Japan; 6 School of Psychological Science, Health Sciences University of Hokkaido, 2-5 Ainosato, Kita-ku, Sapporo, Hokkaido 002-8072 Japan; 7 Sapporo CBT & EAP Center, Goryokai Medical Corporation, Shinoro 8-jo 6-chome 7-6, Kita-ku, Sapporo, Hokkaido 002-8028 Japan

**Keywords:** Sleep, Insomnia, Presenteeism, Systematic review

## Abstract

**Background:**

Sleep problems interfere with work performance. Decreased work productivity due to health problems is defined as presenteeism. Although empirical data on the improvement of presenteeism by sleep interventions have been published, a systematic review elucidating whether there is a difference in the improvement of presenteeism across various types of sleep interventions has not yet been published. This systematic review of studies aimed to clarify which sleep interventions are more likely to be effective in improving presenteeism.

**Methods:**

The electronic databases PubMed, PsycINFO, and MEDLINE were used to perform a literature search (the start and end search dates were October 20, 2019, and March 11, 2020, respectively). A combination of terms such as “employee*,” “sleep,” “insomnia,” and “presenteeism” was used for the search. Both randomized and non-randomized control trials were included in this systematic review.

**Results:**

Six types of sleep interventions were identified, including cognitive behavioral therapy for insomnia (CBT-I), sleep hygiene education, yoga, mindfulness, weight loss program, and changing the color temperature of fluorescent lights in the workplace. Only CBT-I improved both sleep problems and presenteeism compared with a control group. The results of this review also show that there is heterogeneity in the measurement of presenteeism.

**Conclusions:**

The results of this systematic review suggested that CBT-I could be adapted for workers with sleep problems and presenteeism. We discussed whether CBT-I improved both sleep problems and presenteeism compared with other interventions. In addition, methods for measuring presenteeism in future research are proposed.

## Background

Presenteeism is an indicator of productivity loss and is characterized by the loss of productivity due to health problems, even though workers are present at work [1]. For example, presenteeism can be assessed from time management demands, physical demands, mental-interpersonal demands, and output demands [2]. Presenteeism increases the risk of absences in the long term [3, 4]. Presenteeism is not only a predictor of productivity loss but also a predictor of sickness absence due to health problems. To improve presenteeism, it is important to focus on the health status of workers.

Sleep plays an important role in mental and physical health. Individuals with insomnia symptoms had higher rates of physical and psychiatric illnesses and presenteeism than individuals without insomnia symptoms [5]. Insomnia symptoms can cause depressive symptoms [6], suicidal ideation [7], metabolic syndrome [8], and work-related accidental deaths [9]. Those with a risk of insomnia have 5.49-times higher rates of presenteeism than those without risk of insomnia [10]. In a study of the general working population, severe insomnia symptoms were found to intensify presenteeism, regardless of sex [11]. Sleep duration also affects presenteeism; 7–8 h of sleep result in lower presenteeism than less than 6 h or more than 9 h, and the relationship between sleep duration and presenteeism follows a U-shaped curve [12, 13]. Short sleep duration can have effects on the economy and induce losses worldwide [14]. Therefore, improving sleep problems leads to improved presenteeism.

Several previous studies have shown that sleep interventions can improve presenteeism. The sleep medication eszopiclone has been reported to improve presenteeism [15]. Internet-based cognitive behavioral therapy for insomnia (CBT-I) and internet-based sleep hygiene education have been reported to improve presenteeism [16, 17]. Thus, improving sleep problems may have an important role in improving presenteeism.

CBT-I, mindfulness meditation, physical activity, and light therapy have been demonstrated to improve sleep problems in systematic reviews and meta-analyses [18–23]. However, no systematic review or meta-analysis has found that sleep interventions improve functioning in daily life. Although empirical data on the improvement of presenteeism by sleep interventions have been previously published, a systematic review on whether there is a difference in the improvement of presenteeism between the various types of sleep interventions has not yet been published. Sleep and physical and mental health are closely related. Therefore, a systematic review of which sleep interventions improve presenteeism may provide guidance for effective intervention for presenteeism. As such, this study sought to clarify which sleep interventions are more likely to be effective in improving presenteeism using a systematic review of the studies.

## Materials and methods

### Search strategies

This study was conducted in accordance with the Preferred Reporting Items for Systematic Reviews and Meta-Analyses (PRISMA) [24] and A MeaSurement Tool to Assess Systematic Reviews (AMSTAR 2) guidelines [25]. The literature search strategy was based on the literature search terms of a systematic review that examined the effectiveness of workplace health promotion in improving presenteeism [26]. We used a combination of terms such as “employee*,” “sleep,” “insomnia,” and “presenteeism.” The electronic databases PubMed, PsycINFO, and MEDLINE were used to search the literature (the start and end search dates were October 20, 2019, and March 11, 2020).

### Study selection

The inclusion criteria for papers were as follows: (1) the presence of employees, (2) sleep intervention must be performed, (3) sleep problems must be measured, (4) presenteeism must be measured, (5) written in English or Japanese, and (6) publication in a peer-reviewed journal. The selection of studies was carried out by YT, SI, and SA. Papers written in English or Japanese were included in the study since the authors were well-versed in both Japanese and English, but not in other languages.

### Data extraction

Information on subject selection criteria, study design, implementation program, duration, program contents, measurement of sleep problems, measurement of presenteeism, sleep problems outcome results, and presenteeism outcome results were extracted from the studies included in the systematic review. Extraction was carried out independently by YT and SI.

### Study quality assessments

Because this review included randomized (RCTs) and non-randomized control trials (non-RCTs), we used different tools for assessing the risk of bias for RCTs and non-RCTs. The risk of bias was assessed independently by YT and SA using the Cochrane Collaboration’s tool for assessing the risk of bias in RCTs [27]. This tool for assessing the risk of bias was based on (1) random sequence generation, (2) allocation concealment, (3) blinding of participants and personnel, (4) blinding of outcome assessment, (5) incomplete outcome data, (6) selective outcome reporting, and (7) other sources of bias. Other sources of bias addressed differences between groups regarding outcome measures at baseline. The tool evaluates each domain as having a “low risk of bias,” “high risk of bias,” and “unclear risk of bias.” The Risk of Bias In Non-randomized Studies of Interventions tool (ROBINS-I tool) was used to assess the risk of bias in non-RCTs [28]. This tool assesses the risk of bias by (1) bias due to confounding, (2) bias in the selection of participants for the study, (3) bias in the classification of interventions, (4) bias due to deviations from intended interventions, (5) bias due to missing data, (6) bias in the measurement of outcomes, and (7) bias in the selection of the reported results. The tool evaluates each domain and the overall assessment as having a “low risk of bias,” “moderate risk of bias,” “serious risk of bias,” “critical risk of bias,” and “no information.”

## Results

### Search findings

**Fig. 1:**
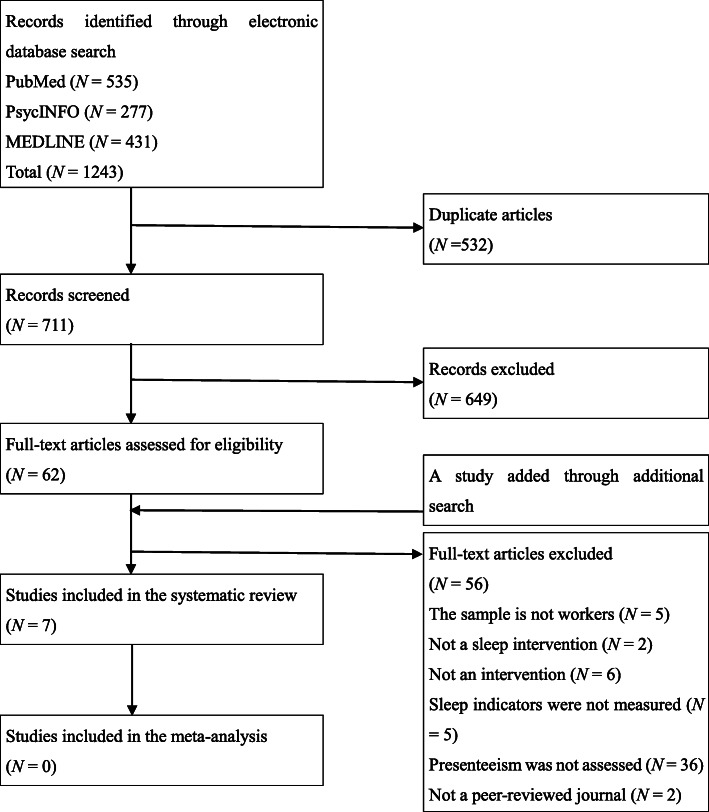
PRISMA flow diagram of the systematic search carried out in this study

Studies included in the review were selected by the first and third authors. Altogether, 1243 studies were screened for duplication. Following the screening, a total of 711 studies were selected, of which 62 studies were selected after evaluating the abstract against the eligibility criteria. YT and SI evaluated the full text of the 62 studies using the eligibility criteria. Finally, six studies were selected for inclusion in this review. One study was added through an additional search. This study was not found by the search formula because “presenteeism” was not included in the abstract. However, the study was included in the review after discussion among the first, second, and third authors who conducted the literature review, because it had been collected in PubMed and met the selection criteria for this study. Finally, seven studies were included in this systematic review. Because of heterogeneity in the measures of sleep problems and presenteeism, we could not perform a meta-analysis (Fig. 1).

### Study quality assessments

The risk of bias was assessed for each of the four RCTs that were included in the systematic review (Table 1). In the domains of blinding of outcome assessment and other sources of bias, 3/4 studies were assessed as having a low risk of bias. Random sequence generation, allocation concealment, and incomplete outcome data indicated a low risk of bias in 2/4 studies. In the domain of blinding of participants and personnel, 1/4 of the studies were determined to have a low risk of bias. There were no reports with a low risk of bias in selective outcome reporting. Moreover, 3/4 of the studies did not include information about the study protocol, which may have caused potential reporting bias.

**Table 1:** Risk of bias items for each included randomized control trial study

	Random sequence generation	Allocation concealment	Blinding of participants and personnel	Blinding of outcome assessment	Incomplete outcome data	Selective outcome reporting	Other sources of bias
Bostock et al. [16]	+	+	?	+	?	?	+
Behrendt et al. [29]	+	+	–	+	–	–	?
Wolever et al. [30]	?	?	?	?	+	?	+
Morgan et al. [31]	–	?	+	+	+	?	+

Low risk of bias is “+”. High risk of bias is “-”. Unclear risk of bias is “?”

The risk of bias was assessed for three non-RCT studies that were included in the systematic review (Table 2). In the domains of bias in the selection of participants for the study and bias in the classification of interventions, all three studies were assessed as having a low risk of bias. In contrast, there were no studies assessed as having a low risk of bias in domains other than bias in the selection of participants for the study and bias in the classification of interventions. In the domains of bias due to missing data and bias in the measurement of outcomes, 2/3 studies were assessed as having a critical risk of bias. Because these domains assessed the post-intervention phase, there may be bias in the post-intervention process.

**Table 2:** Risk of bias items for each included non-randomized control trial study

	Bias due to confounding	Bias in the selection of participants for the study	Bias in classification of interventions	Bias due to deviations from intended intervention	Bias due to missing data	Bias in measurement of outcomes	Bias in the selection of the reported results	Overall
Espie et al. [11]	Serious	Low	Low	Serious	Critical	Critical	Serious	Critical
Burton et al. [17]	Serious	Low	Low	Serious	Critical	Critical	Serious	Critical
Mills et al. [32]	Serious	Low	Low	Serious	Moderate	Moderate	Serious	Serious

In addition, because of the small number of studies included in the review, attention should be paid to the risk of bias of each study in the overall assessment.

### Study characteristics

The characteristics of the studies included in the systematic review are shown in Table 3.

**Table 3:** Characteristics of the studies included in the systematic review

Study	Age	Sex	Country	Job type	Design	Intervention	Duration	Program components
Bostock et al. (2016) [16]	CBT-I 33.9 ± 6.41 years WLC 33.3 ± 5.59 years All 33.6 ± 6.01 years	CBT-I Men: 88 Women: 47 WLC Men: 92 Women: 43	United States	Office-based staff	RCT (vs. WLC)	CBT-I (digital cognitive behavioral therapy: Sleepio)	6 weeks (6 sessions)	Sleep restriction, stimulus control, relaxation, paradoxical intention, belief restructuring, mindfulness
Espie et al. (2018) [11]	50.0 ± 11.14 years	Men: 147 Women: 67	United States	Office Plant Retail & service	Single arm	CBT-I (digital cognitive behavioral therapy: Sleepio)	6 weeks (6 sessions)	Sleep restriction, stimulus control, relaxation, paradoxical intention, belief restructuring, mindfulness
Behrendt et al. (2020) [29]	CBT-I 46.1 ± 9.5 years WLC 46.7 ± 9.7 years All 46.5 ± 9.8 years	CBT-I Men: 29 Women: 59 WLC Men: 32 Women: 57	German	Diverse job categories recruited via media	RCT (vs. WLC)	Internet-delivered CBT-I	6 weeks (6 sessions)	Psychoeducation, sleep hygiene, sleep restriction, stimulus control, relaxation, metacognitive therapy, potential future application
Burton et al. (2016) [17]	20–35 years: 78 36–50 years: 163 ≥51 years: 116	Men: 86 Women: 271	United States	Financial service	Single arm	Sleep hygiene education	5 months	Psychoeducation, sleep hygiene, identification of sleep disorders and information on the sleep disorders, relaxation, mindfulness
Wolever et al. (2012) [30]	Yoga: 41.6 ± 10.1 years Mindfulness: 44.3 ± 9.4 years WLC 42.7 ± 9.7 years	Yoga Men: 24 Women: 66 Mindfulness Men: 22 Women: 74 WLC Men: 10 Women: 43	United States	Insurance	RCT (vs. WLC)	Yoga or mindfulness	Yoga: 12 weeks (12 h) Mindfulness: 12 weeks (14 h)	Yoga Yoga poses, breathing techniques, relaxation, mental techniques Mindfulness Mindfulness meditation
Morgan et al. (2012) [31]	Intervention 44.8 ± 8.3 years WLC 43.7 ± 9.1 years All 44.4 ± 8.6 years	Only men (*N* = 110)	Australia	Aluminum smelter	RCT (vs. WLC)	The workplace power program	14 weeks	Education for weight loss, weekly weight reports, daily diet, and exercise with a pedometer
Mills et al. (2007) [32]	N/A	N/A	United Kingdom	Call-handler	Control trail (2900 K)	Changing the color temperature of fluorescent lights in the workplace	14 weeks	17,000 K vs. 2900 K

*CBT-I* Cognitive behavioral therapy for insomnia, *WLC* Waiting list control, *RCT* Randomized control trial, *N/A* Not applicable

#### Inclusion criteria for participants

In total, 4/7 studies had inclusion criteria for participants, including poor subjective sleep quality, voluntary participation in the program, perceived stress scale score of 10 or more, and body mass index (BMI) in the range of 25 to 40 kg/m^2^. On the other hand, 3/7 studies did not have any selection criteria for participants. In all seven studies, presenteeism was not included in the inclusion criteria for participants.

#### Measurements

Measures of sleep problems included the sleep condition indicator (*N* = 2), insomnia severity index (*N* = 1), Mayo clinic tool (*N* = 1), Pittsburgh Sleep Quality Index (*N* = 1), Epworth sleepiness scale (*N* = 1), and Columbia jet lag scale (*N* = 1).

Measures of presenteeism included the Work Limitation Questionnaire (WLQ; *N* = 3), Work Productivity and Activity Impairment questionnaire (WPAI; *N* = 2), the number of days that work efficiency is reduced due to ill health (*N* = 1), and the World Health Organization Health and Work Performance Questionnaire (WHO-HPQ; *N* = 1).

#### Study designs

With regard to study design, 4/7 studies that were RCTs had a waitlist control. Moreover, 3/7 studies had a non-RCT study design, and 1/3 studies with a non-RCT design had a control group.

#### Effectiveness of sleep interventions

Six different types of sleep interventions were identified, including CBT-I, sleep hygiene education, yoga, mindfulness, weight loss program, and changing the color temperature of fluorescent lighting in the workplace. The type of intervention, outcome measures of sleep problems, outcome measures for and statistical significance of presenteeism, and risk of bias were summarized for each study (Table 4).

**Table 4:** Sleep and presenteeism outcomes for each study included in the systematic review

Study	Intervention	Sleep outcomes	Presenteeism outcomes	Statistical significance (Presenteeism)	Risk of bias (Cochrane Collaboration’s tool)	Risk of bias (ROBINS-I)
Bostock et al. (2016) [16]	CBT-I (Digital cognitive behavioral therapy: Sleepio) Intervention vs. control	SCI	WPAI	*F* (1, 485) = 10.99, *p* < 0.001, *d* = 0.67	Low risk of bias: 4/7 High risk of bias: 0/7 Unclear risk of bias: 3/7	N/A
Espie et al. (2018) [11]	CBT-I (Digital cognitive behavioral therapy: Sleepio) Pre vs. post	SCI	WPAI	*t* (87) = 4.83, *p* < 0.01	N/A	Critical
Behrendt et al. (2020) [29]	Internet-delivered CBT-I Intervention vs. control	ISI	Self-report of the number of days in the past 3 months that work efficiency has decreased due to ill health	Posttreatment N/A 6-months follow-up mean difference between groups = −6.455, *p* < 0.001, *d* = 0.83	Low risk of bias: 3/7 High risk of bias: 3/7 Unclear risk of bias: 1/7	N/A
Burton et al. (2016) [17]	Sleep hygiene education Pre vs. post	Mayo Clinic tool	WLQ Time-management demands Physical demands Mental-interpersonal demands Output demands Overall	No description of the coefficient of the chi-square test Time-management demands *p* < 0.001 Physical demands *n.s.* Mental-interpersonal demands *p* < 0.001 Output demands *p* < 0.001 Overall *p* < 0.001	N/A	Critical
Wolever et al. (2012) [30]	Yoga Mindfulness Intervention vs. control	PSQI	WLQ (overall)	*F* (2, 233) = 2.07, *n.s.*, η^2^ = 0.02	Low risk of bias: 2/7 High risk of bias: 0/7 Unclear risk of bias: 5/7	N/A
Morgan et al. (2012) [31]	The workplace power program Intervention vs. control	ESS	WLQ Time-management demands Physical demands Mental-interpersonal demands Output demands Overall	Time-management demands mean difference between groups is 7.7, *p* = 0.20, *d* = 0.37 Physical demands mean difference between groups is 9.8, *p* = 0.04, *d* = 0.41 Mental-interpersonal demands mean difference between groups is 5.0, *p* = 0.11, *d* = 0.35 Output demands mean difference between groups is 6.1, *p* = 0.23, *d* = 0.29 Overall mean difference between groups is 2.0, *p* = 0.01, *d* = 0.56	Low risk of bias: 4/7 High risk of bias: 1/7 Unclear risk of bias: 2/7	N/A
Mills et al. (2007) [32]	Changing the color temperature of fluorescent lights in the workplace (17,000 K vs. 2900 K) Intervention vs. control Pre vs. post	Item 9 of Columbia Jet Lag Scale (sleepiness during the day)	WHO-HPQ	Overall *t* (67) = −2.72, *n.s.* Intervention change *t* (45) = − 6.07, *p* < 0.001 Control change *t* (22) = − 1.16, *n.s.*	N/A	Serious

*ROBINS-I* Risk of Bias In Non-randomized Studies of Interventions, *CBT-I* Cognitive behavioral therapy for insomnia, *SCI* Sleep Condition Indicator, *WPAI* Work Productivity and Impairment questionnaire, *N/A* Not applicable, *ISI* Insomnia Severity Index, *WLQ* Work Limitation Questionnaire, *n.s.* not significant, *PSQI* Pittsburgh Sleep Quality Index, *ESS* Epworth Sleepiness Scale, *WHO-HPQ* World Health Organization Health and Work Performance Questionnaire

##### Cognitive behavioral therapy for insomnia (CBT-I)

CBT-I was conducted in three studies [11, 16, 29]. Of these, 2/3 studies had an RCT design, and 1/3 studies had a single-arm design. The program included sleep restriction, stimulus control, relaxation, and cognitive reconstruction as common components. All three studies used a weekly program of six sessions, 2/3 studies used the iOS app “Sleepio,” and 1/3 studies used internet-delivered self-help CBT-I. All three studies were internet-based interventions and were not face-to-face.

CBT-I significantly improved insomnia symptoms and presenteeism in all three studies.

##### Sleep hygiene education

Only one study provided sleep hygiene education [17]. The study had a single-arm design. The program included understanding the relationship between sleep and health and productivity, acquiring healthy sleep hygiene habits, identifying and treating sleep disorders, and relaxation and mindfulness. The program was conducted once a month for five sessions. The program was delivered via an internet-based intervention.

Sleep hygiene education significantly improved with insomnia symptoms. On the other hand, significant improvements in presenteeism were observed in time management demands, mental-interpersonal demands, and output demands, except for physical demands.

##### Yoga or mindfulness

Only one study provided a yoga or mindfulness intervention [30]. The study had an RCT design. The yoga intervention included yoga postures, breathing techniques, relaxation, and mental techniques. The mindfulness intervention was mindfulness meditation. Both yoga and mindfulness were conducted for 12 weeks. However, the total duration of yoga was 12 h for 12 weeks and the total duration of mindfulness was 14 h for 12 weeks. Yoga was conducted face-to-face, and mindfulness was conducted face-to-face or via the internet.

Both yoga and mindfulness significantly improved insomnia symptoms, but not presenteeism.

##### Workplace-based weight loss program

Only one study provided a workplace-based weight loss program [31]. The study had an RCT design. The program consisted of weight loss instructions and a pedometer report of activity and diet. The duration of the program was 14 weeks. The program was conducted in the form of both face-to-face and online meetings.

The weight loss program did not significantly improve daytime sleepiness. Only physical demands significantly improved presenteeism, while it was not significantly improved by time management demands, mental-interpersonal demands, and output demands.

##### Changing the color temperature of fluorescent lighting in the workplace

Only one study provided a change in the color temperature of fluorescent lighting in the workplace [32]. The study had a control trial design. The intervention was to change the color temperature of fluorescent lighting on the floor (17,000 K vs. 2900 K).

There was no significant improvement in daytime sleepiness or presenteeism between groups as a result of changing the color temperature of the fluorescent lights (17,000 K vs. 2900 K). However, daytime sleepiness and presenteeism were significantly improved in the 17,000 K group, while the 2900 K group did not show significant improvement in daytime sleepiness and presenteeism in the within-group comparison.

## Discussion

This study sought to identify in a systematic manner which sleep interventions were more likely to improve presenteeism and provide guidelines for effective intervention for presenteeism caused by sleep problems. CBT-I is expected to be highly effective in improving presenteeism. However, this study could not explore the effectiveness of intervention methods for sleep other than CBT-I in improving presenteeism because only a few studies have been conducted to date.

### Heterogeneity in the measurement methods of presenteeism

In this study, a meta-analysis could not be conducted because of the small number of studies included and the use of different methods of measuring presenteeism. Three CBT-I studies were conducted; however, we could not conduct a meta-analysis because of the different measurement methods used in the studies. We were unable to integrate the measures of presenteeism because they were either a standardized self-reported scale or the number of days of reduced work efficiency due to ill health, and integration of these measures would increase heterogeneity and prevent a fair assessment of the quality of the evidence. Heterogeneity in the measurement of presenteeism has also been shown to be a limitation in previous studies [26] that have reviewed the effectiveness of workplace health promotion. The results of this study revealed that there has been no standardized method for measuring presenteeism over the past 10 years. Heterogeneity in the way in which presenteeism is measured arises because of the different definitions of presenteeism in the U.S. and Europe. In the U.S., presenteeism is defined as a decline in work productivity due to workers’ health problems. In Europe, on the other hand, it is defined as people who go to work when they have health conditions that require them to take time off. Each definition has different advantages: the U.S. definition allows us to measure the economic burden of health problems, while the European definition allows for a wide range of research into why people exhibit presenteeism [33]. Future studies will hopefully be able to conduct meta-analyses using both a standardized self-reported scale and the number of days of reduced work efficiency due to ill health as outcome measures and will be able to refine programs to improve presenteeism.

### Effectiveness of sleep interventions

Only CBT-I improved both sleep problems and presenteeism compared with a control group. In other words, CBT-I might be adapted for workers with sleep problems and presenteeism. The results of meta-analyses have suggested that CBT-I is effective in improving insomnia symptoms in the short-term and long-term [18–20]. CBT-I is mediated by work-related rumination and worry in the process of insomnia symptom improvement [29]. In addition, CBT-I not only improves insomnia symptoms, but also improves functional health, psychological well-being, and sleep-related impairment by improving insomnia symptoms [34]. Therefore, CBT-I improves not only insomnia symptoms but also daily life functions impaired by insomnia symptoms. Thus, CBT-I may be effective in improving presenteeism.

The decision to apply CBT-I to workers would need to take into account whether or not they work shifts. Most of the participants of CBT-I in the studies included in the review were office workers. However, it is doubtful whether CBT-I is sufficiently effective in improving insomnia symptoms when administered to shift workers. For example, when shift workers (e.g., nurses, bakers, cabin attendants, security personnel, and land transportation personnel) underwent group CBT-I, self-help CBT-I, and sleep hygiene education, there was no difference in the improvement of insomnia symptoms between groups [35]. Moreover, group CBT-I also did not affect the overall improvement of insomnia in participants whose working areas were stores, offices, warehouses, or logistics workplaces. However, when shift workers (warehouse and logistics) were excluded, there was an improvement in insomnia symptoms [36]. Therefore, the effectiveness of CBT-I for improving presenteeism may be limited to non-shift workers (e.g., office workers) rather than all workers.

CBT-I improved not only subjective insomnia symptoms but also objective sleep onset latency, total wake time, wake time after sleep onset, early-morning awakening, and sleep efficiency [19]. Therefore, interventions that can improve not only subjective sleep indicators but also objective sleep indicators might improve presenteeism.

Sleep hygiene education, mindfulness, yoga, weight loss programs, and changing the color temperature of workplace fluorescent lighting were not effective in improving both insomnia symptoms and presenteeism. These interventions can improve subjective insomnia symptoms, but they do not improve objective insomnia symptoms or are unknown [21, 22, 37, 38]. It remains unclear whether these interventions improve functioning in daily life due to insomnia symptoms. These points are different from CBT-I. Therefore, presenteeism may not have improved. However, since this study included only one study of each intervention, it is not possible to discuss what effect these interventions had on the improvement of presenteeism.

### Inclusion and exclusion criteria for participants

Presenteeism was not included as a criterion for the selection of participants in all of the studies included in this review. This is due to the unclear criterion of presenteeism. The cutoff point for presenteeism is a WHO-HPQ score of 40 points or less for Japanese workers [39]. In the future, the criteria for presenteeism will become clearer as more studies establish cutoff points on existing standardized measures of presenteeism.

One study included in this review had a BMI in the range of 25–40 kg/m^2^ as an inclusion criterion for participants [31]. Being overweight increases the risk of presenteeism [40], but weight loss is not associated with improved presenteeism [41]. However, a study included in this review showed that daytime sleepiness did not improve, but a part of presenteeism did [31]. Therefore, the impact of being overweight on the intervention program is not clear. Thus, having a BMI of 25 or more may be a confounding factor for the intervention effect. Except for weight loss programs, a BMI of 25 or greater should be one of the individual variables to be controlled.

### Study quality assessments

In this study, the risk of bias in RCTs and non-RCTs was assessed separately. The results suggested that RCTs are more likely to have a risk of bias with regard to random sequence generation, allocation concealment, incomplete outcome data, and selective outcome reporting. The results suggested that non-RCTs are more likely to have a risk of bias in response to missing data and selective outcome reporting. These risks of bias can be addressed at the study protocol stage. It is hoped that future RCTs and non-RCTs will provide high-quality interventions by appropriately addressing the areas of potential bias risk identified in this study.

In addition, because of the small number of studies included in the review, attention should be paid to the risk of bias of each study in the overall assessment.

### Limitation

None of the studies included in this review included Asian countries other than Japan. Therefore, generalization of the results of this study to other Asian countries should be made with caution. In particular, Japan has a higher rate of economic loss due to short sleep duration than the United States and Europe [14]. Future, empirical studies are necessary to determine whether sleep interventions can improve presenteeism among Japanese and other Asian populations.

## Conclusion

Although it should be noted that the evidence that CBT-I improves presenteeism remains limited due to the small number of studies included in this study, as well as the inability to conduct a meta-analysis, the results demonstrated that CBT-I might be adapted to daytime workers (e.g., office workers) with sleep problems and presenteeism. Interventions for sleep that improve presenteeism may require improvement in subjective and objective insomnia symptoms and in the functioning of daily life due to insomnia symptoms. Future research is warranted to reveal sleep interventions that can improve not only sleep problems but also daily functioning.

## Data Availability

Not applicable.

## Abbreviations

BMI: Body mass index
CBT-I: Cognitive behavioral therapy for insomnia
RCT: Randomized controlled trial
WHO-HPQ: World Health Organization Health and Work Performance Questionnaire
